# Do We Have Viable Protective Strategies against Anesthesia-Induced Developmental Neurotoxicity?

**DOI:** 10.3390/ijms23031128

**Published:** 2022-01-20

**Authors:** Nemanja Useinovic, Stefan Maksimovic, Michelle Near, Nidia Quillinan, Vesna Jevtovic-Todorovic

**Affiliations:** 1 Department of Anesthesiology, University of Colorado Anschutz Medical Campus, Aurora, CO 80045, USA; stefan.maksimovic@cuanschutz.edu (S.M.); michelle.near@cuanschutz.edu (M.N.); nidia.quillinan@cuanschutz.edu (N.Q.); vesna.jevtovic-todorovic@cuanschutz.edu (V.J.-T.); 2 Neuronal Injury and Plasticity Program, University of Colorado Anschutz Medical Campus, Aurora, CO 80045, USA; 3 Department of Pharmacology, University of Colorado Anschutz Medical Campus, Aurora, CO 80045, USA

**Keywords:** neonatal anesthesia, neuroprotection, neurotoxicity, mitochondria, ROS, signaling pathways, neuroactive steroids

## Abstract

Since its invention, general anesthesia has been an indispensable component of modern surgery. While traditionally considered safe and beneficial in many pathological settings, hundreds of preclinical studies in various animal species have raised concerns about the detrimental and long-lasting consequences that general anesthetics may cause to the developing brain. Clinical evidence of anesthetic neurotoxicity in humans continues to mount as we continue to contemplate how to move forward. Notwithstanding the alarming evidence, millions of children are being anesthetized each year, setting the stage for substantial healthcare burdens in the future. Hence, furthering our knowledge of the molecular underpinnings of anesthesia-induced developmental neurotoxicity is crucially important and should enable us to develop protective strategies so that currently available general anesthetics could be safely used during critical stages of brain development. In this mini-review, we provide a summary of select strategies with primary focus on the mechanisms of neuroprotection and potential for clinical applicability. First, we summarize a diverse group of chemicals with the emphasis on intracellular targets and signal-transduction pathways. We then discuss epigenetic and transgenerational effects of general anesthetics and potential remedies, and also anesthesia-sparing or anesthesia-delaying approaches. Finally, we present evidence of a novel class of anesthetics with a distinct mechanism of action and a promising safety profile.

## 1. Introduction

Since its invention in the mid-nineteenth century, general anesthesia (GA) has been an indispensable component of surgical procedures. Conquering the pain and providing comfort were important milestones allowing for the performance of progressively longer and more invasive procedures. Modern anesthesia protocols and techniques have been traditionally considered safe and beneficial in many pathological settings. However, over the past three decades, we have been grappling with the harsh realization that GA might not be harmless to the developing brain. Preclinical evidence is overwhelming—the notion that GA causes detrimental and long-lasting injury to the developing brain has been confirmed by hundreds of preclinical studies in numerous animal species [1,2,3,4,5,6,7,8,9,10]. Alarmingly, the growing body of clinical evidence is also pointing towards similar cognitive-affective abnormalities in humans exposed to GA during the early years of their lives [11,12,13,14,15,16].

How do we address these growing concerns that GA may pose a substantial healthcare burden in years to come considering that millions of children are being anesthetized every year? Over the past several decades a considerable effort has been made to devise neuroprotective strategies that would enable the use of currently available GAs while avoiding their unwanted neurotoxic effects. Based on our present knowledge of the cellular pathways that play a role in neuronal and glial demise, we and others have reported many different approaches using numerous compounds that were introduced with a goal to control, prevent or protect against the deleterious effects of GAs. Thus far, there are hundreds of preclinical studies that have examined different types of neuroprotective strategies. Here, we provide a summary of select strategic approaches with a primary focus on different mechanisms of neuroprotection and potential clinical applicability.

## 2. Neurotoxicity of General Anesthetics

Early work by Ikonomidou and colleagues have reported the rapid and widespread apoptotic injury and brain mass reduction in rat pups following administration of ethanol [17], the oldest known anesthetic to humankind with both the NMDA-blocking and GABA-mimetic properties, much like virtually all clinically used anesthetics that induce hypnosis via alterations in GABAergic and glutamatergic neurotransmission. Presumably, GAs that act via NMDA-inhibition (nitrous oxide, ketamine) [18] deprive the developing neurons of important glutamatergic survival signaling which can lead to apoptosis [19]. On the other hand, high neonatal expression of the Na^+^-K^+^-2Cl^−^-1 (NKCC1) importer renders GABA excitatory in early life [20]. Later developmental upregulation of the K^+^-2Cl^−^-2 (KCC2) chloride extruder pump reverses the Cl^−^ gradient and enables the inhibitory action of GABA [20]. GABA-mimetic GAs (e.g., midazolam, propofol, etomidate, isoflurane, sevoflurane, desflurane) [18] may disturb this developmental trajectory by increasing the NKCC1/KCC2 ratio [21], which could be the mechanism of neurotoxicity induced by these agents.

Anesthesia-induced neuroapoptosis is widespread and profound, with almost all brain regions affected, with the rate of apoptosis increased up to 70-fold compared to the age-matched baseline [1,2,22,23]. Ultrastructurally, neuronal loss is reflected by devastated and empty-looking neuropil, containing nothing but distorted debris [24]. The neuronal cytoplasm contains numerous swollen and/or degenerated mitochondria [24,25], indicative of deranged fusion and fission [26]. The neurons which survive anesthesia treatment exhibit signs of significant synaptic derangement [24]. Neurons become hyperexcitable [25,27,28], and long-term potentiation, the synaptic surrogate for learning and memory, has been repeatedly reported to be suppressed [1,29,30,31,32]. Unsurprisingly, these changes often persist through adulthood and manifest as deficits in multiple behavioral domains [1,9,33,34,35,36,37,38,39].

## 3. Neuroprotective Strategies

Identification of key derangements in intracellular signal transduction pathways and well-established histo-morphological and behavioral findings have supported the implementation of targeted therapeutic approaches aiming to protect against deleterious effects of early-life anesthesia. These neuroprotective strategies will be categorized based on the cellular targets known to be instrumental in GA-induced developmental neurotoxicity (Table 1).

Although the majority of studies have mainly centered on neurons as primary targets for anesthetic neurotoxicity and neuroprotection, thus focusing on neurons as the primary culprit for lasting behavioral deficits, we are becoming increasingly aware of the importance of glial activation and destruction after neonatal GA exposure [40,41,42]. However, due to very complex interactions between the neurons and glia, especially during the early stages of brain development, it has been difficult to draw definitive conclusions as to the relative contributions of neurons versus glial cells in ensuing functional derangements following GA. To account for this limitation in the field of anesthesia-induced neurotoxicity, we have centered the discussion of neuroprotective strategies on neurons as primary targets for neuroprotective interventions, while keeping in mind that glia deserves more attention in future studies.

**Table 1:** Preclinical studies examining neuroprotective effects of various drugs in response to general anesthetics. s.c., subcutaneously; PND, postnatal day; i.p., intraperitoneally; iso, isoflurane; PKC, protein kinase C; Nrf2, nuclear factor E2-related factor 2; ROS, reactive oxygen species; MDA, malondialdehyde; SOD, superoxide dismutase; GPx, glutathione peroxidase; sevo, sevoflurane; ROS, reactive oxygen species; CFC, contextual fear conditioning; MWM, Morris water maze; BDNF, brain-derived neurotrophic factor; PI3K, phosphatydilinositol 3 kinase; CAT, catalase; ATP, adenosine triphosphate; NF-kB, nuclear factor kappa B; IL-6, interleukin 6; TNF-α, tumor necrosis factor alpha; RAM, radial arm maze; N_2_O, nitrous oxide; pAkt, phosphorylated Akt; GSK-3β, glycogen synthase kinase 3 beta; EPO, erythropoietin; NGF, neural growth factor; NOR, novel object recognition; DEX, dexmedetomidine; MAPK, mitogen-activated protein kinase; ERK, extracellular signal-regulated kinase; pERK1/2, phosphorylated extracellular signal-regulated kinase 1/2; i.v., intravenously; MCP-1, monocyte chemoattractant protein-1; CCR2, chemokine receptor type 2; SGZ, subgranular zone; SVZ, subventricular zone; NsTyr, N-stearoyl-L-tyrosine; HDAC, histone deacetylase; HAT, histone acetyltransferase; CBP, CREB-binding protein; NMDA. N-methyl-D-aspartate; ↑, increased/upregulated/improved; ↓, decreased/downregulated/worsened.

Study	Drug Regimen	Species	Anesthesia Regimen	Mechanism	Drug Effects
Mitochondrial stability and ROS					
Yon 2006 [43]	Melatonin 1, 5, 10, 20 mg/kg s.c.	Rat, PND7	Triple cocktail * for 2, 4 or 6 h	Mitochondrial stabilization	↓cortex and anterior thalamus neuroapoptosis ↑Bcl-x_L_ ↓Cytochrome c
Li 2018 [44]	Melatonin 10 mg/kg i.p.	Rat, PND7	1.5% iso for 4 h	PKC/Nrf2 activation	↓hippocampal neuroapoptosis ↓mitochondrial damage ↓ROS, MDA; ↑SOD, GPx
Ji 2015 [45]	Curcumin 20 mg/kg i.p.	Mouse PND6–8	3% sevo 2 h daily, 3 consecutive days	ROS scavenging	↑Freezing (CFC) ↓Escape latency (MWM) ↓Cortex and hippocampus neuroapoptosis ↑BDNF
Bai 2013 [46]	Resveratrol 50, 100, 200 µM for 24 h	Primary neurons	2% iso for 6 h	PI3K/Akt activation	↓neuroapoptosis ↑mitochondrial stability ↑CAT, SOD, ATP, Ca^2+^
Tang 2021 [47]	Resveratrol 100 mg/kg daily for 6 days, i.p.	Mouse PND6–8	3% sevo for 2 h daily for 3 consecutive days	↓inflammation	↓NF-kB, IL-6, TNF-α ↓microglial activation ↓Escape latency (MWM)
Boscolo 2013 [33]	Pramipexole 1 mg/kg four doses, i.p.	Rat PND7	Triple cocktail * for 6 h	N/A	↑learning and memory (MWM) Females more than males
Boscolo 2012 [48]	Pramipexole 1 mg/kg four doses, i.p.	Rat PND7	Triple cocktail * for 6 h	Mitochondrial stabilization ROS scavenger	↓subiculum neuroapoptosis ↓mitochondrial damage ↑learning and memory (RAM)
Liu 2013 [49]	L-carnitine 1, 30, 100 µM for 24 h	Primary neurons	10 µM ketamine for 24 h	ROS scavenging	↓ROS ↓neuroapoptosis Preserves neuronal morphology
Yan 2014 [50]	L-carnitine 30 µM (culture) 300 mg/kg (rats)	Rat PND7–9; Primary neurons	10 µM ketamine (cultures) 75 mg/kg for 3 consecutive days (rats)	↓inflammation ↓ROS	↓hippocampal neuroapoptosis ↓ROS, proinflammatory factors ↑learning and memory (MWM, passive avoidance test)
Zou 2008 [51]	L-carnitine 50–500 mg/kg i.p.	Rat PND7	75%N_2_O + 0.55% iso for 2, 4, 6, 8 h	Mitochondrial stabilization	↓cortex neuroapoptosis Normalized Bax/Bcl-x_L_ ratio
Ma 2016 [52]	α-lipoic acid 100 mg/kg i.p.	Rat PND7	2.5% sevo for 2 h	↑PI3K/Akt ↓GSK-3β	↓hippocampal neuroapoptosis ↑learning and memory (MWM)
Zhao 2018 [53]	α-lipoic acid 5 µM	Primary neurons	4% desflurane for 2–96 h	Mitochondrial stabilization ↓ROS ↑PI3K/Akt	↓neuroapoptosis ↑SOD, ↑pAkt normalized Bax/Bcl-2 ratio
**Signal transduction pathways**					
Tsuchimoto 2011 [54]	EPO 50,000 IU/kg s.c.	Mouse PND7	1% iso for 6 h	N/A	↓dentate gyrus neurodegeneration ↑learning and memory (MWM)
Pellegrini 2014 [55]	EPO 5000 IU/kg i.p.	Rat PND7	2% sevo for 6 h	↑BDNF ↑NGF	↓cortex neuroapoptosis ↑object recognition (NOR) ↑learning and memory (MWM)
Lv 2017 [56]	DEX 25–75 µg/kg i.p.	Rat PND7	100 mg/kg propofol	↑PI3K/Akt	↓hippocampal neuroapoptosis ↑pAkt ↑pGSK-3β
Li 2014 [57]	DEX 25–75 µg/kg i.p.	Rat PND7	0.75% iso for 6 h	↑PI3K/Akt	↓hippocampal neuroapoptosis normalized Bad/Bcl-x_L_ ratio
Liu 2013 [58]	Lithium 5 × 120 mg/kg over 6 h, i.p.	Rat PND7	5 × 20 mg/kg ketamine over 6, i.p.	↑PI3K/Akt	↓neuroapoptosis ↑pAkt ↑pGSK-3β ↓cyclin D1
Straiko 2009 [59]	Lithium 6 mEq/kg, i.p.	Mouse PND5	40 mg/kg ketamine, s.c., or 50 mg/kg propofol, i.p.	↑MAPK/ERK	↓cortex and caudate/putamen neuroapoptosis ↑pERK1/2
Zhong 2006 [60]	Lithium 10 mg/kg, i.p.	Mouse PND7; Primary neurons	2 × 2.5 mg/kg ethanol, s.c.	?? not PI3K/Akt	↓widespread neuroapoptosis ↓primary neuronal death
Noguchi 2016 [61]	Lithium 0.15–0.75 mEq/kg, i.v.	Rhesus, PND6	1.5–3% iso for 5 h	↑MAPK/ERK??	↓neurons and oligodendrocyte apoptosis
Wang 2018 [62]	Minocycline 2 × 30 mg/kg, s.c.	Mouse PND5	2 × 2.5 mg/kg ethanol, s.c.	↑PI3K/Akt	↓thalamus, cortex, cerebellum neuroapoptosis ↓IL-6, MCP-1, CCR-2 ↑pAkt, pGSK-3β
Ren 2019 [63]	Minocycline 2 × 30 mg/kg s.c.	Mouse PND5	2 × 2.5 mg/kg ethanol, s.c.	↑PI3K/Akt ↑MAPK/ERK	↓spinal cord neuroapoptosis ↓MCP-1, IL-6 ↑pAkt, pGSK-3β, pERK1/2
Giri 2018 [64]	Minocycline 40 mg/kg, i.p.	Rat PND7	9 mg/kg midazolam, i.p.	N/A	↑SGZ and SVZ neurogenesis ↑learning and memory (MWM)
Lu 2017 [65]	Minocycline 40 mg/kg, i.p.	Rat PND7	40 mg/kg ketamine, i.p.	↑PI3K/Akt	↑SGZ and SVZ neurogenesis ↑pAkt, pGSK-3β ↑learning and memory (MWM)
Wang 2013 [66]	NsTyr 10 mg/kg (rats); 1 µM (culture)	Rat, PND7; Primary neurons	3% sevo for 2, 4, 6, 8 h	↑MAPK/ERK Mitochondrial stabilization	↓neuroapoptosis ↑pERK1/2, ↑Bcl-2 ↑Learning and memory (MWM)
**Steroid hormones**					
Li 2014 [67]	17β-estradiol 600 µg/kg, s.c.	Rat PND7	75 mg/kg ketamine for 3 h consecutive days, i.p.	↑BDNF ↑PI3K/Akt	↑cortex neuroapoptosis ↑pAkt, ↑BDNF ↑learning and memory (MWM)
Lu 2006 [68]	17β-estradiol 3 × 300 µg/kg, s.c.	Rat PND7	Triple cocktail * 2, 4, 6 h	↑PI3K/Akt??	↓thalamus and cortex neuroapoptosis
Asimiadou 2005 [69]	17β-estradiol 300–900 µg/kg i.p.	Rat PND7 Primary neurons	phenobarbital/phenytoin (50 mg/kg) MK801 (0.5 mg/kg	↑MAPK/ERK ↑PI3K/Akt Estrogen receptors?	↓neuroapoptosis ↑pAkt, ↑pERK 1/2
Li 2019 [70]	17β-estradiol 3 × 100 µg/kg i.p. (rats) 100 nM for 24 h (cultures)	Rat PND7; Primary neurons	ketamine: 40 mg/kg i.p. (rats) 100 µM for 24 h (cultures)	GSK-3β inactivation	↑learning and memory (MWM) ↑proliferation, ↓apoptosis (cultures) ↓pGSK-3β
Yang 2021 [71]	Testosterone	Rat PND6	3% sevo, 2 h daily for 3 consecutive days	GSK-3β inactivation	↑endogenous brain testosterone ↓tau phosphorylation ↓learning and memory (MWM)
**Epigenetic changes**					
Dalla Massara 2016 [72]	sodium butyrate 1.2 g/kg i.p. (rats) 5 mM for 24 h (cultures)	Rat PND7 Primary neurons	Triple cocktail * for 6 h (rats) or 24 h (cultures)	HDAC inhibition	↑histone H3 acetylation ↑number of neurons and dendritic branches ↓mIPSC half-width and decay
Joksimovic 2018 [73]	entinostat 10 mg/kg i.p.	Rat PND7	Triple cocktail * for 6 h	HDAC inhibition	↑histone H3 acetylation normalization of mIPSC freq.
Zhong 2016 [74]	swimming exercise 4 × 5 min for 4 weeks	Mouse PND7–9	0.75% iso, 4 h daily for 3 consecutive days	↑HAT?? ↓HDAC??	↑Freezing (CFC) ↑histone acetylation ↑hippocampal CBP
**Anesthetic-sparing**					
Cattano 2008 [75]	Xenon 70% for 4 h	Mouse PND7	0.75% iso for 4 h	NMDA antagonism	↓cortex and caudate/putamen neuroapoptosis when combined with iso
Gill 2021 [76]	Xenon 0, 35, 70% for 6 h	Rat PND8	2.7% sevo alone 1.8% sevo + 35% xenon 0.9% sevo + 70% xenon for 6 h	NMDA antagonism?	↓acidosis ↓hippocampus and cortex neuroapoptosis (70% xenon)
Ma 2007 [77]	Xenon 30, 60, 75% xenon ± iso for 6 h	Rat PND7	0.75% iso ± xenon for 6 h	Mitochondrial stabilization	↓hippocampal neuroapoptosis ↓caspase-3 and -9 ↓cytochrome C
Shu 2010 [78]	Xenon 70% for 2 h pretreatment	Rat PND7	70% N_2_O + 0.75% iso for 6 h	Mitochondrial stabilization	↓hippocampus and cortex neuroapoptosis ↑Bcl-2, ↓cytochrome c, p53 ↑Freezing (CFC)

* Triple cocktail contains midazolam (9 mg/kg, i.p.), followed by 6 h of N_2_O (75%) and isoflurane (0.75%).

### 3.1. Mitochondrial Stability and Reactive Oxygen Species (ROS) Scavenging

Mitochondria play the central role in GA-induced neuroapoptosis [22]. The mitochondria-dependent pathway is controlled by a Bcl-2 family of proteins with both pro-apoptotic and anti-apoptotic roles. Bcl-2 and Bcl-x_L_, the mitochondrial “gate keepers”, compete with cytosolic Bax and Bid, the mitochondrial “gate openers”, to maintain mitochondrial membrane integrity [79,80]. The increased ratio of pro-apoptotic versus anti-apoptotic proteins (e.g., Bax/Bcl-2 ratio) in response to cellular stress causes instability of mitochondrial membranes [79,81]. This impairs their integrity, and makes them leaky and prone to rupturing which, in turn, leads to cytosolic exudation of cytochrome c and activation of executioner caspase-3 [80], which is responsible for neuronal demise [82]. GAs are known to modulate several Bcl-2 proteins leading to mitochondrial damage [22,43,51,53,55,57] and the activation of the intrinsic mitochondrial apoptotic pathway [22].

Importantly, GAs are also known to increase the production of reactive oxygen species (ROS) [33,48,83]. ROS are highly reactive free radicals which, if left unchecked, cause damage by indiscriminately oxidizing lipids, proteins and DNA [84]. Nuclear factor E2-related factor 2 (Nrf2) is a transcriptional factor controlled by cellular kinases such as pAkt and PKC (protein kinase C) [85,86]. Upon activation, Nrf2 induces expression of genes coding for ROS-scavenging enzymes (e.g., glutathione peroxidase, catalase, superoxide dismutase) [87,88].

Based on the growing knowledge of molecular underpinnings of anesthesia-induced developmental toxicity, we and others have proposed the use of several safening strategies that may curtail the activation of mitochondrial apoptotic pathways and excessive ROS production. Three naturally occurring phytochemicals (melatonin, curcumin and resveratrol) have emerged as beneficial neuroprotective agents and are also highly effective and versatile antioxidants. The mechanisms by which these chemicals reduce ROS-mediated cellular damage are threefold: via direct scavenging and neutralization of free radicals; indirectly via stimulation of intracellular protective systems such as GPx, CAT and SOD [89,90]; and via the upregulation of the anti-apoptotic Bcl-2 family of proteins [43]. For example, melatonin administration prevents neuronal death induced by a triple anesthetic cocktail of midazolam, nitrous oxide and isoflurane in a dose-dependent fashion by increasing Bcl-X_L_ expression [43], reducing cytochrome c release and inhibiting the mitochondrial-dependent apoptotic pathway. It was subsequently shown that melatonin administration prevents isoflurane-induced ROS damage in the hippocampus by activation of the PKCα/Nrf2 pathway [44].

Similarly, the antioxidant and anti-inflammatory properties of curcumin were highlighted in a study showing that curcumin prevented neuroapoptosis and long-term memory impairment resulting from 3% sevoflurane exposure in neonatal mice [45]. Finally, resveratrol effectively protected against damage induced by in vitro and in vivo administration of isoflurane [46] and sevoflurane [47], respectively. Interestingly, the neuroprotective effects of resveratrol were lost in the presence of specific anti-Akt siRNA, which suggests that activation of the PI3K/Akt pathway might mediate much of the neuroprotective effects of resveratrol [46]. 

Antioxidative phytochemicals are generally considered safe and devoid of significant side effects related to their use while also having good blood-brain barrier penetrability. Due to the confounding presence of underlying disease in real clinical scenarios, the hypnotic, analgesic, anxiolytic and anti-inflammatory properties of melatonin [91] may be of additional value if given perioperatively. 

Another type of promising strategy relies on pharmacological agents that confer direct mitochondrial protection and mitochondrial membrane stabilization, and indirect protection on ROS scavenging. Such groups of agents include, but are not limited to, pramipexole (PPX) and L-carnitine. PPX is a non-ergot dopaminergic receptor agonist that is FDA approved for the treatment of Parkinson’s disease. It is known to have a high affinity for mitochondrial uptake and, hence, it is often found in concentrations that are about eight-fold higher compared to the ones detected in the cytosol [92]. Our previous work has shown that PPX not only protects mitochondria from GA-induced ‘leakiness’ by ROS scavenging and maintaining the integrity of the inner mitochondrial membrane [33,48], but also blocks developmental neuroapoptosis [48] while preventing the development of GA-induced cognitive impairments when administered around the time of GA exposure [33,48]. 

Another promising mitochondrial ‘protector’ is a naturally occurring compound, L-carnitine, which functions as a transport shuttle for long-chain free fatty acids into the mitochondria where they undergo β-oxidation [93]. Notably, L-carnitine is a mitochondrial stabilizer that neutralizes buildup of toxic acyl-CoA during β-oxidation [94], although it also serves as a direct ROS scavenger [94]. The neuroprotective effects of L-carnitine in the context of GA exposure were first noted in cultured primary rat forebrain neurons [49]. The addition of L-carnitine to culture medium prevented ketamine-induced ROS formation and DNA damage. When rat pups were exposed to combined nitrous oxide and isoflurane anesthesia, L-carnitine pretreatment 24 h and 30 min prior to anesthesia stabilized the mitochondrial membrane which, in turn, reduced the Bax/Bcl-2 ratio resulting in downregulation of neuroapoptosis [51]. Importantly, it was shown that the long-term memory deficits caused by neonatal ketamine exposure can be prevented by L-carnitine pre-administration [50]. Although L-carnitine shows promising results in preventing damage induced by neonatal anesthesia, these findings should not be misconstrued as universally applicable. The paradoxical increase in ROS production, inflammation and hepatotoxicity, as well as disturbances in renal function, were associated with L-carnitine use [95], and should further be carefully evaluated before L-carnitine is recommended as a protective agent against GA-induced developmental neurotoxicity.

The importance of ROS formation, lipid peroxidation and protein oxidation in GA-induced neuronal damage during synaptogenesis is further confirmed by the fact that α-lipoic acid, a known antioxidant, acting via both direct scavenging and by replenishment of intracellular antioxidants [96], is associated with particularly strong neuroprotective properties in preclinical models. Namely, when α-lipoic acid was given to neonatal rats around the time of sevoflurane administration, there was almost a complete reversal of sevoflurane-induced caspase-3 activation and memory impairment [52]. The authors concluded that neuroprotection by α-lipoic acid was achieved by the reactivation of the PI3K/Akt pathway and phosphorylation-inactivation of GSK-3β [52]. The in vitro apoptosis of primary hippocampal neurons induced by desflurane was also reversed by α-lipoic acid [53], through ROS scavenging, superoxide dismutase (SOD) reactivation and decrease in the Bax/Bcl-2 ratio. The overall effects of α-lipoic acid in these studies were related to favoring pro-survival signaling pathways and the deactivation of ROS produced in response to an early exposure to GA.

So far, there are no reports of any significant adverse effects following α-lipoic acid treatment [97]. Overall, the observed neuroprotective effects of α-lipoic acid, combined with its safety profile, make this an appealing ‘safening’ agent. Since α-lipoic acid gained interest as a potential neuroprotectant only recently, more preclinical evidence is needed to support its use.

### 3.2. Modulation of Intracellular Signal-Transduction Pathways

**Figure 1:**
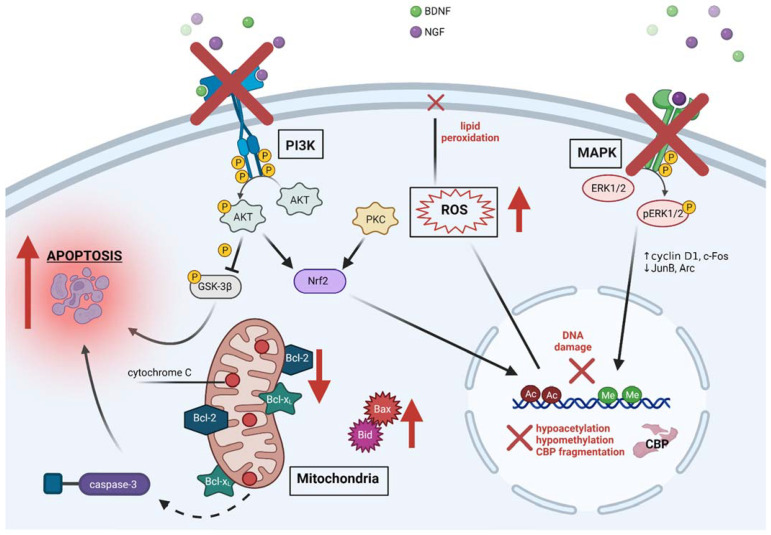
Summary of key cellular targets in the pathophysiology of anesthesia-induced developmental neurotoxicity. Red arrows and crosses represent the main events by which GAs exert their deleterious effects in immature neurons. GAs deprive neurons of survival signals initiated by growth factors, transduced by two major kinase pathways. Furthermore, GAs may directly induce ROS formation, genetic and epigenetic disturbances, and mitochondrial instability leading to cytochrome c exudation and apoptosis. Black borders indicate neurotoxicity-reversal points, triggered by neuroprotective strategies discussed previously. BDNF, brain-derived neurotrophic factor; NGF, neural growth factor; PI3K, phosphatidylinositol 3 kinase; MAPK, mitogen-activated protein kinase; PKC, protein kinase C; ERK, extracellular signal-regulated kinase; GSK-3β, glycogen synthase kinase 3 beta; ROS, reactive oxygen species; Nrf2, nuclear factor E2-related factor 2; Ac, acetylated histones; Me, methylated DNA; CBP, CREB-binding protein.

GAs cause dysregulation of evolutionarily conserved and highly regulated signal transduction pathways which are of critical importance for early development and survival of neurons [98,99]. Normally, growth factors such as brain-derived neurotrophic factor (BDNF) and neural growth factor (NGF) cause phosphorylation and activation of two major kinase pathways: PI3K/Akt [100] and MAPK/ERK [99]. Phosphorylated Akt (pAkt) further phosphorylates and inactivates glycogen synthase kinase 3 beta (GSK-3β) which induces apoptosis via an unknown mechanism if left unopposed [101,102]. Phosphorylated ERK (pERK) translocates to the nucleus, induces transcription of genes associated with survival (*cyclin D1*, *c-Fos*), and suppresses expression of cell cycle inhibitors (*JunB, Arc*) [103]. The deleterious effects of GAs are summarized in Figure 1.

When the role of BDNF and NGF was assessed in the setting of GA-induced developmental neurotoxicity, erythropoietin (EPO) was considered potentially promising. Although EPO is mainly known for its effects on erythropoiesis, EPO has recently gained attention due to its non-erythropoietic neuroprotective properties indicated by heightened fetal neuroapoptosis in EPO receptor-deficient mice [104]. Importantly, EPO was successful at abolishing the memory impairment induced by 6 h of isoflurane anesthesia in neonatal mice [54]. In order to elucidate the mechanism of EPO-mediated neuroprotection, Pellegrini and colleagues administered EPO immediately at the end of 6 h sevoflurane exposure [55] and found that EPO increases levels of BDNF and NGF which together with a decreased Bax/Bcl-2 ratio [55] suggests modification of survival signaling pathways as a result of growth factor binding to their respective receptors. Although valuable for our understanding of the role of survival signaling pathways and growth factors, it was observed that EPO administration to preterm or low-birth infants conferred increased risk of neutropenia and of retinopathy of prematurity [105]. Therefore, the neuroprotection by EPO administration might be limited due to its questionable safety profile in its target population.

When the activation of two major kinase pathways (PI3K/Akt [100] and MAPK/ERK [99]) was examined, several protection strategies were considered. For example, dexmedetomidine (an α_2A_- adrenergic receptor agonist successfully used as adjuvant to more potent intravenous and inhaled GAs), a clinically used anaesthetic with sedative and analgesic properties, is rapidly gaining recognition as a promising neuroprotective agent [106]. Due to its imidazoline structure, it is possible that some of the beneficial effects of dexmedetomidine may be conferred by the activation of imidazoline I2 receptors [107]. The anti-apoptotic effects of dexmedetomidine were documented following administration with propofol and isoflurane in rat pups [56,57,108]. Dexmedetomidine reversed neurotoxic effects of general anesthetics via modulation of the PI3K/Akt pathway; i.e., the pAkt level was increased [56,57] while GSK-3β was phosphorylated and inactivated [56]. Furthermore, dexmedetomidine treatment led to a reduction of the Bid/Bcl-2 ratio, shifting the balance in favor of anti-apoptotic members of the Bcl-2 protein family [57]. However, the initial high level of enthusiasm for dexmedetomidine was somewhat hampered by the reports showing that high doses of dexmedetomidine resulted in paradoxical worsening of GA neurotoxicity [109,110] and a dose-dependent increase in mortality [111]. One potential explanation is that the α_2_-receptor selectivity is lost with increasing doses of dexmedetomidine [112], therefore, the toxic effects of high dexmedetomidine are, in fact, due to its α_1_-agonism. As stated earlier, since dexmedetomidine is already administered perioperatively as an adjuvant due to its sedative-hypnotic and analgesic effects [113], the attention it has received by the scientific community is warranted and, as such, requires further evaluation.

Another promising strategy may involve minocycline, a semi-synthetic tetracyclic antibiotic with beneficial effects outside of its antibacterial properties. Minocycline is a highly effective microglial inhibitor [114] with well-documented anti-inflammatory, anti-apoptotic and neuroprotective roles. It exerts neuroprotective effects via modulation of PI3K/Akt pathways [115]. In neonatal mice, minocycline reversed ethanol-induced neuroapoptosis in the brain and spinal cord [62,63] and induced phosphorylation-inactivation of GSK-3β, thereby inhibiting microglial activation and production of proinflammatory cytokines. When administered before midazolam [64] or ketamine [65], minocycline improved performance in the Morris water maze spatial memory paradigm and promoted neurogenesis in subventricular and subgranular hippocampal zones.

Due to its ability to block microglial activation and the ability to restore protective PI3K/Akt pathways, as well as its good blood-brain barrier permeability [114], minocycline might be one of the prime candidates for future studies focused on mitigation strategies aimed at protecting an immature brain from general anesthetics.

Another promising modulator of PI3K/Akt [100] and MAPK/ERK [99] signaling pathways is a clinically used drug, lithium. In rat pups treated with ketamine, lithium administration was shown to increase phosphorylation-activation of Akt and phosphorylation-inhibition of GSK-3β with greater than a three-fold reduction in caspase-3 positive profiles compared to ketamine alone [58]. Several studies have suggested that lithium confers protection against ketamine/propofol [59] and ethanol [60] via Akt-independent mechanisms via upregulation of pERK1/2. Preclinical evidence deemed lithium to be very effective at abolishing neuronal apoptosis induced by different neurotoxins and GAs in both rodents and non-human primates [58,59,60,61]. In a dose-dependent fashion, lithium treatment strongly blocks neuroapoptosis—in fact, Straiko and colleagues reported that 6 mEq/kg lithium administered together with ketamine or propofol to neonatal mice resulted in activated caspase-3 levels that were indistinguishable from controls [59]. Despite being a very powerful blocker of GA-induced developmental neuroapoptosis, it is noteworthy that lithium has substantial side effects [116] and causes numerous drug interactions [117]. Further studies should be performed to determine whether lithium can be safely and reliably used to prevent GA-induced developmental neurotoxicity.

Although better studied in the field of medicinal use of cannabinoids, N-stearoyl-L-tyrosine (NsTyr) is gaining interest as a potential ‘safening’ agent. Due to its structural homology to anandamide, an endocannabinoid, NsTyr has a unique mechanism of action via binding to CB_1_ cannabinoid receptors, which is believed to serve neuroprotective purposes [118,119]. For instance, a study by Wang and colleagues [66] has shown that neuronal apoptosis and memory deficits induced by 3% sevoflurane were abolished in rats pretreated with NsTyr. The putative mechanism of NsTyr-neuroprotection stems from the increased levels of Bcl-2 and pERK1/2. Notably, the neuroprotective effects of NsTyr were completely lost when MAPK/ERK pathways were blocked [66]. It is noteworthy that, to date, this is the only performed study to evaluate the neuroprotective effects of NsTyr in rat pups exposed to anesthesia. Although promising, further studies should be conducted to assess the efficacy and safety of NsTyr. 

### 3.3. Steroid Hormones

Over the past decade, the steroid sex hormones have been considered up-and-coming neuroprotective agents. In support of this notion, several studies have documented the anti-apoptotic effects of 17β-estradiol after neonatal exposure to anesthesia. By increasing the level of pAkt, 17β-estradiol decreased neuroapoptosis and improved learning and memory in neonatal rats treated with ketamine [67] and a triple cocktail of midazolam, nitrous oxide and isoflurane [68]. Other studies suggested that another effect of 17β-estradiol, namely an increase in the level of pERK1/2 [69] and phosphorylation-inactivation of GSK-3β [70], might be responsible at least in part for the neuroprotective effects of sex hormones.

The latest study by Yang and colleagues has shown that exogenous administration of testosterone attenuated sevoflurane-induced cognitive impairment in male neonatal mice. Using a multitude of approaches, it was concluded that low levels of testosterone—a physiological occurrence in neonatal male pups—could be a culprit for their vulnerability to sevoflurane-induced cognitive impairment [71] as administration of exogenous testosterone to male neonatal mice reduced tau phosphorylation and prevented cognitive impairment by inhibiting the GSK-3β activation [71].

These studies raised the question as to whether the exogenous steroids can be used as a viable neuroprotective strategy during vulnerable stages of brain development. Having said that, concerns about the safety of steroid use in the context of neonatal brain pathology were raised when increased neuroapoptosis was observed in hippocampus and dentate gyrus of rats treated with 17β-estradiol in combination with muscimol, a selective GABA_A_ agonist [120]. Similarly, exogenous testosterone pretreatment followed by isoflurane anesthesia in neonatal female rats worsened performance in memory tasks later in life and testosterone injections were associated with increased NKCC1 and decreased KCC2 transporters compared to controls [121]. These studies emphasize the importance of recognizing gender differences and maintaining the fine balance between different neurotransmitters, especially GABA, during critical stages of brain development. 

### 3.4. Modulation of Epigenetic Modifications

The tightness of DNA packaging inside nucleosomes, comprised of DNA strands wound up around histone proteins, is regulated via application of chemical tags which loosen up or make a structure tighter. Two forms of chemical tags—methylation and acetylation—modify the accessibility of genes to transcription factors in diametrically opposite directions. Epigenetic modifications change these chemical tags and alter the tension within nucleosomes without altering the DNA sequence.

Acetylation of histones by histone acetyltransferases tends to a relaxed chromatin structure and an increase in the expression of genes. There is evidence to suggest that initial insult in memory impairment by administration of a triple anesthetic cocktail (nitrous oxide, midazolam and isoflurane) is via fragmentation of CREB-binding protein (CBP) [72]. Since CBP contains histone acetyltransferase activity [122], anesthesia exposure indirectly causes hypoacetylation of H3 histone and down-regulation of BDNF and c-Fos, which play critical roles in memory formation and consolidation [72,122]. 

On the contrary, DNA methylation on cytosine carbon 5 makes genes less accessible to transcription factors. It has been shown that rat pups exposed to 6 h of sevoflurane exhibited a significant hypomethylation in the subiculum and upregulated JunB and Arc protein levels [123]. Because methylation status is stable and inheritable, it has been reported that the hypomethylated pattern was passed on to offspring that were never anesthetized [123]. 

Fortunately, histone deacetylase inhibitors such as entinostat [73] and sodium butyrate [72] provide promising and effective reversal of GA-induced H3 histone hypoacetylation. In addition to pharmacological intervention, exercise has shown to be beneficial. For example, rats subjected to four weeks of regular swimming exercise showed signs of memory improvement, increased acetylation of H3 and H4, and increased c-Fos and CBP in the hippocampus [74]. The fact that swimming exercise, conducted after the injury, improves the phenotype raises hope and calls for further studies focused on similar non-pharmacological approaches that may improve behavioral phenotypes in GA-naïve offspring of animals exposed to GAs as neonates. 

### 3.5. Anesthetic-Sparing Strategy

In addition to dexmedetomidine utility as an adjuvant to currently used general anesthetics as stated earlier, another anesthetic that has come to the forefront as a potentially beneficial accompaniment is xenon. Considering that GAs exert a neurotoxic effect in dose-dependent fashion with higher doses and/or multiple exposures being particularly detrimental to normal behavioral and cognitive development resulting in an increased risk for learning disabilities [14], this leads one to question whether anesthetic-sparing strategies could result in less deleterious effects of currently used GAs.

Xenon is a gas anesthetic with a minimal alveolar concentration (MAC) of 92% in the pediatric population [124]; hence, it cannot be used as a sole anesthetic under normobaric conditions but rather could serve as an anesthetic-sparing agent. Xenon causes hypnosis via NMDA-inhibition, but through a distinct mechanism, by competing with glycine at the receptor co-activation site [113]. It has paradoxical effects on neuronal death: when given at 1 MAC, it triggers neuroapoptosis of similar magnitude to the equipotent concentration of isoflurane or sevoflurane [75,125]. However, when given as an adjuvant to isoflurane or sevoflurane, xenon was shown to (1) decrease the requirement of other GAs needed to achieve the surgical plane of anesthesia [75,76], and (2) reduce caspase-3 immunoreactivity, a hallmark of GA-induced developmental neuroapoptosis [75,76,77,78]. Furthermore, perioperative administration of xenon has a number of advantages, including cardiovascular stability, analgesia and rapid recovery [113]. Combining xenon with sevoflurane was shown to be safe for children under the age of 4 who were undergoing diagnostic cardiac catheterization [124]. Since xenon is produced by fractional distillation of liquefied air, the major downsides of xenon use are its scarcity in the atmosphere and its production cost [126]. 

### 3.6. The Timing of GA Exposure and Regional Anesthesia

The timing of GA exposure. Paracelsus said that “all things are poisonous and nothing is without poison; only the dose makes a thing not poisonous.” In the case of GA-induced developmental neurotoxicity, we have realized that not only the dose, but also the timing of exposure is important. The “window of vulnerability” to GAs correlates with the period of brain development marked by intense synaptogenesis and axonal pruning, two crucial elements of proper formation of fundamental neuronal networks we will rely on for the rest of our lives. Since the “window of vulnerability” is relatively well-defined, a tempting proposal would be to delay GA beyond this period, therefore potentially circumventing the neurotoxicity concerns. Unfortunately, the conundrum of GA use in very young children is much more complex. As discussed at the latest Surgical Panel of PANDA symposium [127], it seems highly unlikely that delaying procedures due to risk of not-yet well-defined deficits later in life would outweigh the immediate benefits of corrective or life-saving surgery. Although surgical experts agreed that the total dose and duration of anesthesia should be kept as low as reasonably achievable, so far there is little incentive or scientific merit for delaying surgeries as a viable protective strategy.

Regional (spinal) anesthesia. Although the indications are largely confined to surgical procedures involving the lower part of the body, spinal anesthesia might be an important substitute to GA in select clinical scenarios. While the available data advise caution with regard to local anesthetics causing neuronal damage [128], preclinical evidence so far has disproven the hypothesis of heightened neuroapoptosis following spinal anesthesia in rat neonates [129,130,131]. Furthermore, two clinical studies have demonstrated the feasibility and safety of spinal anesthesia for early-life surgical procedures [132,133]. Although the surgical procedures were minor and of relatively short duration, these studies suggested no residual neurobehavioral deficits when assessed later in life. Spinal anesthesia might also confer additional benefits such as the lower incidence of hemodynamic, respiratory and gastrointestinal complications perioperatively compared to GA [134]. Although the evidence remains scarce, it implies that regional anesthesia might be a safer option with regard to long-term neurobehavioral sequelae, and further studies are needed to determine its value as a neuroprotective strategy. 

## 4. Development of Alternate General Anesthetics

With that in mind, the ultimate question becomes whether we are at the point to seriously contemplate the development of new GAs that would be a safe alternative to currently used ones, the GAs that would lack the neurotoxic potential. At present, developmental neurotoxicity seems to be a class-wide property of virtually all clinically used GAs. Their common feature is that they induce hypnosis by acting as either NMDA-blockers (e.g., ketamine, nitrous oxide) [18] or GABA-mimetics (propofol, midazolam, isoflurane, desflurane, sevoflurane) [18]. Therefore, an intriguing solution to this conundrum would be to develop novel anesthetics with different cellular targets.

Potentially attractive cellular targets are low voltage-gated calcium channels, so called T-channels known to get activated at low membrane potential and, as such, are important for synaptic neurotransmission and control of neuronal excitability [135]. A class of agents referred to as neuroactive steroid analogs have been shown to share the hypnotic properties of propofol and ketamine but to preferentially modulate T-channels. One such analog is 3β-OH [(3β,5β,17β)-3-hydroxyandrostane-17-carbonitrile], a hypnotic neuroactive steroid shown to have blocking properties for T-channels at hypnotic brain concentrations [136], but without a direct effect on either GABA_A_ or NMDA ligand-gated channels. Importantly, 3β-OH induces a surgical plane of anesthesia similar to ketamine but, unlike ketamine, it didn’t cause observable neuroapoptosis in the subiculum, thalamus and cingulate cortex of rat pups during their most vulnerable age [136]. Importantly, 3β-OH anesthesia was not associated with memory deficits when examined later in life [136].

Two other promising neuroactive steroid analogs, alfaxalone and CDNC24, were evaluated for their neurotoxic potential in vivo [137]. Like propofol, these compounds provided a similar level of anesthesia, but unlike propofol they did not induce neuroapoptosis in rat pups [137]. Possible mechanisms suggested not only a direct blocking effect on T channels but also a decrease in presynaptic spontaneous release of GABA [136,137]. Given that GABA plays an excitatory role during early development [20,21], neuroactive steroids might protect immature neurons against GABA-mediated excitotoxic injury and provide a possible explanation as to why neuroactive steroids do not trigger neuroapoptosis during early stages of development.

## 5. Concluding Remarks and Future Directions

With the overwhelming preclinical evidence, there should be very little doubt that GA exposure in early life causes substantial impairments, morphologically and functionally. Indeed, an excellent meta-analysis by Lin and colleagues reported that published preclinical studies (until year 2016) reporting structural damage outnumbered those with negative results by a 12:1 ratio [109]; those which detected alterations in functional outcomes outnumbered those which did not by a ratio of over 4:1 [138]. Faced with such formidable odds, it would be imprudent, in our opinion, to assume that humans are somehow uniquely insusceptible to GA-induced developmental neurotoxicity. 

Despite the outstanding preclinical evidence, clinical studies have so far remained equivocal. While some trials may suggest significant long-term functional impairments following early-life anesthesia [11,12,13,14,15,16], several large studies failed to document long-term sequelae in various neurological domains following childhood anesthesia [132,139,140]. Ambiguous outcomes in an attempt to translate compelling and overwhelming preclinical evidence to the human population could be attributable, at least in part, to different types of biases that inherently plague clinical trials, especially when it comes to the long-term follow up of socio-emotional, cognitive and behavioral development that spans decades in humans and could be influenced by many confounders. Additionally, humans have a much larger window of vulnerability compared to rodents [141], making comparisons between age at exposure and anesthesia duration difficult. Finally, after more than twenty years of intense research, the field of anesthetic neurotoxicity still struggles with the lack of standardized animal models and tools for neurobehavioral assessment [142], and undertaking steps towards resolving these conflicts would be of tremendous value for future research.

With this notion in mind, are we ready to embrace the strategies that would enable us to use GAs while avoiding their damaging effects on a very young brain? If so, what should be considered the most promising and, as such, the most translatable strategies? It is encouraging to learn about the vast preclinical evidence reporting different forms of protective strategies. While this review is not intended to provide an exhaustive summary of all possible strategies, it offers a cross-section of potential approaches that could perhaps be adopted in the near future, although presently none of the strategies are without their own downsides. Having said that, we should consider whether the time has come for a change in the nihilistic approach of ignoring potential consequences of an early-life anesthesia while insisting that co-morbidities and/or surgical interventions are the sole culprit. Are we prepared to take ownership for some of the reported outcomes? Only then will we be free to forge ahead in devising even safer ways of anesthetizing our youngest, sickest and most vulnerable members of society. There is no doubt that we are up to the challenge. The question is, ‘Are we ready to embrace it?’

## Data Availability

Not applicable.
